# Diagnostic dilemma in a neonate: Pseudo Hypoaldosteronism mimicking congenital adrenal hyperplasia

**DOI:** 10.12669/pjms.42.2.12705

**Published:** 2026-02

**Authors:** Hamid Bin Tariq, Suleman Saeed, Ahmed Arslan, Saima Batool

**Affiliations:** 1 Hamid Bin Tariq Final Year MBBS Student, University College of Medicine and Dentistry, Lahore, Pakistan; 2 Suleman Saeed Final Year MBBS Student, University College of Medicine and Dentistry, Lahore, Pakistan; 3 Ahmed Arslan Post Graduate Resident Paediatric, University of Lahore Teaching Hospital, Lahore, Pakistan; 4 Saima Batool, MBBS, FCPS (Pediatrics), MME. University of Lahore Teaching Hospital, Lahore, Pakistan

**Keywords:** Pseudo hypoaldosteronism, Congenital Adrenal Hyperplasia, Adrenal Gland Hyperplasia, Hyperkaliemia, Hyponatremia, Renin, Aldosterone, Metabolic Acidosis

## Abstract

Pseudo hypoaldosteronism (PHA) and congenital adrenal hyperplasia (CAH) can present with similar biochemical abnormalities in neonates, making early diagnosis challenging. We report a neonate who presented with hyponatremia, hyperkalemia, and metabolic acidosis and was initially managed as CAH with steroid therapy.

**Outcome:**

Despite treatment, the electrolyte abnormalities persisted, prompting further evaluation. Genetic testing revealed a homozygous pathogenic mutation in SCNN1B, confirming autosomal recessive PHA Type I-B.

**Conclusion:**

Persistent electrolyte derangements despite appropriate steroid therapy should raise suspicion for PHA. Early genetic testing is essential for accurate diagnosis and targeted management in neonates with overlapping presentations of CAH and PHA.

## INTRODUCTION

Pseudo hypoaldosteronism and congenital adrenal hyperplasia (CAH) are unique endocrinological diseases that can present with similar and overlapping clinical features, making a final diagnosis much more difficult. CAH is relatively common in the pediatric population, whereas PHA is a much rarer disorder. Pseudo hypoaldosteronism (PHA) is a condition in which patients present with adrenal insufficiency with normal to elevated levels of aldosterone. This arises from resistance to aldosterone at the renal tubular level. Patients present with hyponatremia, hyperkalemia, and metabolic acidosis.^1^ PHA can be classified as either Type-I (inherited) Type-II (acquired). Renal Type-I PHAs is mostly autosomal dominant, but can also occur with an autosomal recessive pattern due to mutations of the mineralocorticoid receptor, leading to less binding of aldosterone to its receptors.^2^ Patients with Type-II PHA present with hyperkalemia, hypertension, hyperchloremia, metabolic acidosis, variable levels of aldosterone, and decreased renin values.^3^

Congenital adrenal hyperplasia is an autosomal recessive disorder that is due to mutations of genes that are needed to produce enzymes involved in the synthesis of glucocorticoids, aldosterone, and androgens, leading to adrenal glands hyperplasia.^4^ CAH has several forms; each form comes with a mutation of a different enzyme. The most common is 21-alpha hydroxylase deficiency.^5^ Depending on the form and enzyme deficiency, patients can have varied presentations, such as hyponatremia, hypovolemia, hypertension, hyperkalemia, metabolic acidosis, hyperpigmentation, shock, hypoglycemia, secondary sexual dysfunctions, infertility, and ambiguous genitalia.

Early differentiation between CAH and PHA is crucial, as misdiagnosis can lead to inappropriate treatment, worsening electrolyte imbalance, adrenal crisis, or preventable morbidity and mortality. Prompt recognition ensures timely initiation of condition-specific therapy.

This case report presents a pediatric patient with a diagnostic dilemma between CAH and PHA, with some features inclining towards PHA, while the remaining ones point towards CAH. Therefore, this case report is being presented to emphasize the necessity for detailed clinical evaluation and appropriate diagnostic testing to ensure correct management.

## CASE PRESENTATION

The patient, a male neonate referred to as Baby XYZ, was born at the University of Lahore Teaching Hospital (ULTH) on the 7th of July, 2024. With a strong immediate cry, a good Apgar scores and normal blood sugar levels, the patient’s birth was otherwise uneventful. The patient received routine immunization and was exclusively breastfed. However, on the 7th day of life, the patient presented with jaundice, lethargy and poor feeding, leading to readmission.

Upon admission, the patient exhibited stable vital signs, including a heart rate of 116 bpm, a respiratory rate of 42 breaths per minute, a temperature of 98°F, and an SpO_2_ of 97%. Weight and anthropometrics were appropriate for age. Physical examination indicated the presence of jaundice extending to the lower limbs, with fair neonatal reflexes and normal muscle tone.

Initially, the infant was treated for breastfeeding jaundice, presenting with a serum total bilirubin (STB) level of 16 mg/dL. Phototherapy was initiated, and intravenous fluids were administered. After 24 hours of treatment, a decrease in STB levels was observed, and discharge plans were made. However, the mother reported ongoing feeding difficulties, necessitating further observation. On the ninth day, Baby XYZ’s condition worsened; he became lethargic, experienced respiratory distress, showed prolonged capillary refill time, diminished neonatal reflexes, and weak peripheral pulses.

A comprehensive evaluation was conducted. The CBC and RFTs results were normal. The ABG analysis revealed the presence of metabolic acidosis. The serum electrolyte report demonstrated severe hyperkalemia and hyponatremia. Despite the implementation of initial management strategies, including phototherapy, the hyperkalemia, hyponatremia and metabolic acidosis persisted, indicating a potential adrenal disorder. Diagnostic considerations were primarily centered on pseudo hypoaldosteronism and congenital adrenal hyperplasia. To rule out these differentials, it was recommended to assess serum electrolytes, 17-hydroxyprogesterone, plasma renin, serum aldosterone, and urinary sodium levels. The results are summarized in Table-I and II.

**Table-I:** Initial Clinical and Laboratory Parameters at Presentation.

Parameter	Timing of Test	Result	Reference Range	Interpretation
Serum Total Bilirubin (STB)	7th day of life	16 mg/dL	0.2–1.1 mg/dL	Elevated (neonatal jaundice)
Sodium (Na^+^)	9th day of life	127 mEq/L	135–145 mEq/L	Hyponatremia
Potassium (K^+^)	9th day of life	10.3 mEq/L	3.5–4.5 mEq/L	Severe hyperkalemia
Urinary Sodium	Initial	55.4 mmol/L	54–190 mmol/L	Normal
Serum Cortisol	Follow-up	10.10 µg/dL	4.82–19.5 µg/dL	Normal
Plasma ACTH	Follow-up	40.9 pg/mL	<46 pg/mL	Normal
Serum DHEA-SO_4_	Follow-up	281 µg/dL	80–560 µg/dL	Normal

**Table-II:** Serial hormonal profile showing evolution of renin, aldosterone and 17-hydroxyprogesterone levels.

Parameter	Initial	Follow up	Reference Range	Interpretation
17-Hydroxyprogesterone (17-OHP)	4.342 ng/mL	19.55 ng/mL	0.00–8.0 ng/mL	Elevated
Plasma Renin Activity	18.98 uIU/mL	>500 uIU/mL	4.4–46.1 uIU/mL	Markedly Elevated
Serum Aldosterone	64.5 ng/mL	>100 ng/mL	2.52–39.2 ng/mL	Markedly Elevated

These findings are suggestive of pseudo hypoaldosteronism (PHA). Given that the laboratory findings did not support CAH, hydrocortisone and fludrocortisone were discontinued, and symptomatic treatment continued. Following the cessation of salt therapy, serum potassium levels increased again. Steroid therapy was initially started due to concern for salt-wasting CAH, where glucocorticoid and mineralocorticoid replacement is the standard of care. Once early hormonal studies did not support CAH, steroids were withdrawn to avoid unnecessary exposure, and the patient was managed symptomatically with salt supplementation. However, the recurrence of hyperkalemia after stopping salt therapy prompted reconsideration of both differentials, leading to re-initiation of steroids while further testing was arranged. After a week, additional laboratory tests, including plasma renin, serum aldosterone, 17-OH progesterone, ACTH levels, serum cortisol, and DHEA-SO4 were recommended. Abdominal and pelvic ultrasound showed normal findings, except for bilaterally prominent adrenal glands suggestive of adrenal hyperplasia. Laboratory results revealed normal plasma ACTH levels and serum cortisol levels.

Serum DHEA-SO_4_ was also within the normal range. However, repeat testing revealed a significant elevation in 17-hydroxyprogesterone along with markedly increased aldosterone and plasma renin activity. Therapeutically, hyperkalemia was treated by discontinuing potassium supplementation in IV fluids and administering calcium gluconate to stabilize cardiac function. Sodium bicarbonate with insulin and Kayexalate were utilized to facilitate potassium transport. To mitigate the risk of adrenal insufficiency, hydrocortisone and fludrocortisone were also initiated for controlling acidosis. Although initial improvement was observed, the infant’s electrolyte levels remained unstable after cessation of salt therapy, raising concerns regarding possible PHA.

**Fig.1:**
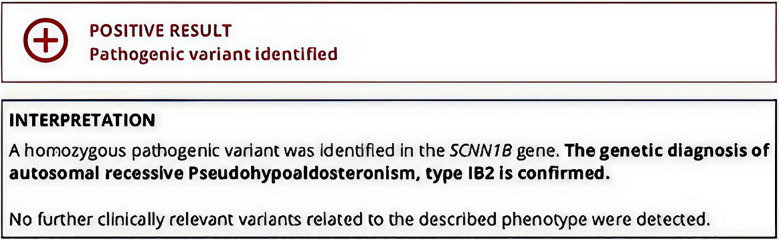
Report for Genetic Testing Screenshot of the genetic testing report showing homozygous pathogenic variant in the *SCNN1B* gene, consistent with autosomal recessive Pseudo hypoaldosteronism type IB2. No additional clinically relevant variants were identified.

Following a consultation with a pediatric endocrinologist, it was determined that the working diagnosis of PHA Type-I appeared more probable; however, CAH could not be conclusively ruled out without conducting genetic testing.^6^ The recommendation was made to maintain the ongoing medical treatment. Arrangements were established for genetic testing and ACTH stimulation test to be performed at six months. The results of genetic testing suggested a homozygous pathogenic variant in the SCNN1B gene, and a diagnosis of autosomal recessive Pseudo hypoaldosteronism Type-IB2 was made (Fig.1).

## DISCUSSION

The most crucial point that is highlighted in our case is the lack of clinical improvement despite steroid therapy, leading to a clinical complexity in differentiating CAH and PHA. This is especially relevant in neonates or infants when electrolyte disturbances, particularly hyperkalaemia, remain uncorrected despite stress dose corticosteroids and fluid resuscitation, which should ideally show significant improvements in patients with congenital adrenal hyperplasia. This diagnostic overlap is particularly challenging in neonates, as early laboratory values such as 17-OHP and renin may not yet be significantly elevated, leading to potential misclassification. The evolution of hormonal markers serves as an important diagnostic clue in distinguishing evolving CAH from aldosterone resistance states.

Initially, congenital adrenal hyperplasia with 21-hydroxylase deficiency was considered due to the presentation of electrolyte disturbances, metabolic acidosis, and clinical deterioration. This form of CAH results in abnormal synthesis of cortisol and aldosterone, giving rise to a compensatory increase in ACTH and precursor hormones like 17-hydroxyprogesterone. However, normal 17-OHP levels along with normal ACTH and cortisol initially made CAH less likely. Nonetheless, levels of 17-OHP did eventually rise, suggesting that the timing of testing may influence diagnostic interpretation.

Concurrently, the patient displayed features that were more consistent with Type-IB PHA, which is an autosomal recessive form requiring lifelong treatment and is caused by pathogenic variation of the ENaC subunit genes.^7^ The diagnosis of PHA was supported by increased levels of aldosterone, initially normal renin and 17-OPH levels, and recurrence of electrolyte derangements upon withdrawal of salt therapy. Another factor that favored PHA in our case was the lack of hyperpigmentation.

The ENaC channel is found in all aldosterone-responsive organs including kidneys, colon, lungs and sweat glands.^8^ Coupled with the basolateral sodium-potassium ATPase, ENaC directly reabsorbs sodium along with indirectly facilitating secretion of potassium.^3^ As a result, mutations in this channel may cause failure to thrive, hypovolemia, hypertension, hyperkalemia and metabolic acidosis.^3^

In comparison to the case series presented by Babiker et al., which calls attention to the diagnosis of four infants with pseudo hypoaldosteronism and the common diagnostic and management obstacles encountered, our case report presents a single case analysis that further emphasizes the diagnostic difficulties when differentiating Pseudo hypoaldosteronism from Congenital Adrenal Hyperplasia.^1^ When Babiker et al. highlights the importance of serum aldosterone dilution and adjustments in management of PHA, our case focuses on the initial diagnostic dilemma and presents an extensive hormonal workup followed by genetic testing, which ultimately confirmed the diagnosis. To further highlight the diagnostic complexity, the key biochemical differences between salt-wasting CAH and PHA are summarized in Table-III.

**Table-III:** Comparison of biochemical and Clinical Features of Salt-Wasting Congenital Adrenal Hyperplasia and Pseudo hypoaldosteronism.

Parameter	Salt-Wasting CAH	PHA Type I
17-OHP	High	Normal or mildly ↑ initially
Aldosterone	Low or low-normal	Very high
Renin	High	Very high
Sodium	Low	Low
Potassium	High	Very high, persistent
Response to Steroids	Good (rapid improvement)	Poor/No response
Pathophysiology	Cortisol/aldosterone synthesis defect	Aldosterone resistance (ENaC mutation)
Pigmentation	May show hyperpigmentation	Absent

## CONCLUSION

Persistent hyperkalemia unresponsive to steroid therapy should alert clinicians to possible PHA. A multidisciplinary approach involving endocrinology and genetics can ensure accurate diagnosis and prevent unnecessary steroid exposure.

### Authors’ Contribution:

**HT SS:** Literature search, manuscript writing and editing.

**AA:** Data collection. Critical Review

**SB:** Literature search, Critical Review.

All authors reviewed and approved the final manuscript. All authors take full responsibility for the accuracy and integrity of the work and agree to be accountable for all aspects of the manuscript.
